# Early identification of sarcopenia in patients with diabetes mellitus combined with osteoporosis: development and validation of a gender-specific nomogram

**DOI:** 10.3389/fendo.2025.1590247

**Published:** 2025-04-30

**Authors:** Mingzhong Yu, Yunyun Su, Ping Wang, Min Pan

**Affiliations:** ^1^ Department of Geriatrics, The First Affiliated Hospital, Fujian Medical University, Fuzhou, China; ^2^ Department of Geriatrics, National Regional Medical Center, Binhai Campus of the First Affiliated Hospital, Fujian Medical University, Fuzhou, China; ^3^ The First Clinical Medical College, Fujian Medical University, Fuzhou, China; ^4^ Department of Comprehensive Ward, The First Affiliated Hospital, Fujian Medical University, Fuzhou, China

**Keywords:** sarcopenia, T2DM, osteoporosis, nomogram, early screening

## Abstract

**Objective:**

The aim of this study was to develop a predictive model to screen for sarcopenia in patients with type 2 diabetes mellitus (T2DM) combined with osteoporosis, with a view to identifying and intervening early in those at high risk of falls and fractures, thereby reducing the risk of disability and death in the elderly.

**Methods:**

Clinical data collection, physical performance evaluations, and dual-energy X-ray absorptiometry were performed on 847 patients with T2DM combined with osteoporosis. Risk factors for sarcopenia were identified using the least absolute shrinkage and selection operator method. Furthermore, a sex-specific nomogram was constructed based on these indicators to predict the occurrence of sarcopenia, and the predictive efficacy and clinical value of the model were evaluated by receiver operating characteristic curve and decision curve analysis.

**Results:**

The prevalence of sarcopenia in patients with T2DM combined with osteoporosis was 33.88%, with men having a significantly higher prevalence than women. Among male patients, body mass index, 25-hydroxyvitamin D, and calcium levels were associated with a decreased risk of sarcopenia, whereas age and weight-adjusted waist index were associated with an increased risk. In female patients, body mass index and creatine kinase were associated with a decreased risk of sarcopenia, while age, weight-adjusted waist index, and low-density lipoprotein cholesterol were associated with an increased risk. The area under the receiver operating characteristic curve of the nomogram was 91.2% in males and 84.5% in females, showing high predictive accuracy.

**Conclusions:**

In this study, gender-specific nomograms were successfully established, which provided an effective tool for early screening of sarcopenia in patients with T2DM combined with osteoporosis. These models help healthcare professionals identify individuals at high risk of falls and fractures, facilitating timely preventive measures and reducing the burden on the social healthcare system.

## 1 Introduction

Sarcopenia is an age-related loss of muscle mass accompanied by a decline in muscle strength and function, often coexisting with chronic diseases such as type 2 diabetes mellitus (T2DM) and osteoporosis, which may lead to an increased risk of falls and fractures. The diagnosis of sarcopenia is usually based on measurements of muscle mass, assessments of muscle strength, and tests of physical performance.

With the acceleration of global population aging, the prevalence of three chronic diseases, T2DM, osteoporosis and sarcopenia, is increasing (1–3). Patients with T2DM are at a high risk of developing osteoporosis and sarcopenia. Insulin resistance, microangiopathy, and oxidative stress caused by T2DM may be involved in the pathophysiological processes of osteoporosis and sarcopenia (4, 5). Additionally, common risk factors such as aging, metabolic abnormalities, and physical inactivity contribute to the comorbidity of osteoporosis and sarcopenia (6). A study in the UK reported a prevalence of sarcopenia of up to 50% in postmenopausal women with osteoporosis (7). Another study in elderly Europeans showed that patients with sarcopenia had a four-fold higher risk of osteoporosis compared to those without sarcopenia (8). The triad of comorbidity of T2DM, osteoporosis and sarcopenia is particularly common in the elderly. These conditions greatly increase the risk of falls, fractures, and subsequent disability and death (6), posing not only a serious threat to individual health, but also a heavy burden on the social healthcare system. Therefore, it is crucial to promptly identify this high-risk group and provide early intervention.

Despite the relatively clear diagnosis of osteoporosis, the diagnostic criteria and screening methods for sarcopenia remain controversial and challenging (9). The complexity of muscle function assessment, limitations of muscle mass measurement equipment, and geographic variability in diagnostic criteria make it difficult to generalize the diagnosis. There is also a lack of effective screening tools for early identification of sarcopenia risk in patients with T2DM combined with osteoporosis, resulting in a large number of high-risk elderly patients who do not receive adequate attention and intervention.

This study aims to fill this gap by establishing a gender-specific nomogram to predict the risk of sarcopenia in patients with T2DM combined with osteoporosis. Nomograms play an increasingly important role in modern medical research and clinical practice as tools that simplify complex statistics and provide intuitive predictive graphics. Our goal is to provide an early screening tool to help healthcare professionals identify high-risk patients so that timely preventive measures can be taken to reduce the incidence of falls and fractures, and to understand and manage patients with comorbidities of T2DM, osteoporosis and sarcopenia from a new perspective.

## 2 Methods

### 2.1 Study population

A total of 859 patients with T2DM combined with osteoporosis, hospitalized at the First Affiliated Hospital of Fujian Medical University from 1 March 2014 to 31 December 2022, were included in this cross-sectional study. Inclusion criteria consisted of hospitalized patients with a confirmed diagnosis of T2DM (10) and osteoporosis (11). Exclusion criteria included patients with neuromuscular diseases or other endocrine disorders affecting muscle mass and function, acute infectious diseases, severe organ dysfunction such as those of the heart, lungs, and kidneys, malignant tumors, psychiatric disorders or cognitive dysfunction, and patients who used drugs affecting muscle mass or bone density during the study period. We excluded patients with missing data on body weight (n=2), blood counts (n=4), and renal function (n=6), resulting in a final sample of 847 subjects. All participants voluntarily signed an informed consent form after being fully informed about the study, which adhered to the principles of the Declaration of Helsinki. The study was approved by the Ethics Committee of the First Affiliated Hospital of Fujian Medical University (approval number: [2021] 336).

### 2.2 Clinical data collection, laboratory testing and body composition analysis

All participants underwent detailed clinical data collection at the time of enrollment, including but not limited to age, gender, disease history, drug use history, and lifestyle habits. Additionally, all patients underwent a comprehensive physical examination, including measurements of weight, height, waist circumference, blood pressure, heart rate, grip strength, and walking speed, as well as the necessary laboratory tests, such as complete blood glucose, glycated hemoglobin, blood routine, biochemical tests and bone metabolism-related indicators.

### 2.3 Body mass index and weight-adjusted-waist index

BMI is a metric calculated by dividing body weight in kilograms by the square of height in meters, commonly used in clinical practice to assess body fat and categorize weight status. WWI is calculated by dividing waist circumference (cm) by the square root of body weight (kg) to more accurately reflect the relationship between abdominal fat and body weight.

### 2.4 Grip strength and gait speed measurement

Grip strength was measured using an electronic hand dynamometer (model EH101, Senssum, Hubei, China). All participants were asked to stand up straight with the dynamometer in their hands and their arms parallel to their body without leaning. Participants were allowed to perform the test three times with both their right and left hands, with the highest value being the final result. Gait speed was measured using a 4-meter walk test, where participants were instructed to walk at their normal pace for more than 6 meters from a standing position to avoid deceleration before reaching the 4-meter mark. The test duration was recorded using an electronic counter (XJ894, Xin Jie, Zhejiang, China).

### 2.5 Body composition analysis

Body composition, including upper limb muscle mass, upper limb fat mass, lower limb muscle mass, lower limb fat mass, trunk muscle mass, and trunk fat mass, was determined using dual-energy x-ray absorptiometry (DEXA, Lunar Prodigy scanner, GE Lunar Corporation, Madison, WI, USA). The Appendicular Skeletal Muscle Index (ASMI) evaluates skeletal muscle mass relative to body size and is calculated by dividing the combined muscle mass of the upper and lower limbs by the square of the height. The formula is ASMI (kg/m2) = muscle mass of the limbs (kg)/height (m)2.

### 2.6 Definition of sarcopenia

Muscle strength (grip strength) and physical performance (usual gait speed) were used as screening tests according to the Asian Working Group for Sarcopenia 2019 definition. Low grip strength was defined as less than 28 kg for males and less than 18 kg for females, while low gait speed was defined as less than 1.0 m/s. Screening tests were followed by DEXA, with the ASMI cut-off point for the diagnosis of sarcopenia was < 7.0 kg/m2 for males and < 5.4 kg/m2 for females (12).

### 2.7 Statistical analyses

All statistical analyses were performed using SPSS version 25.0 and R version 4.1.1. Continuous variables were expressed as mean ± standard deviation or median (interquartile range), based on the distribution of the data. Categorical variables were expressed as frequencies and percentages. Comparisons between groups were conducted using the Student’s t-test for normally distributed continuous variables, the Mann-Whitney U test for non-normally distributed continuous variables, and the Chi-square test for categorical variables. Spearman’s correlation analysis was used to assess the correlation between risk factors and different muscle content areas. For random missing values in the data, the Multiple Imputation technique was used to enhance data completeness and analysis accuracy. A P-value of < 0.05 was considered statistically significant for all tests.

The samples were randomly divided into 7:3 training and validation cohorts using the “sample()” function from the base package in R. And least absolute shrinkage and selection operator (LASSO) regression analyses were performed using the “glmnet” package in R to identify key predictor variables. Candidate variables were screened by maximizing the log-likelihood ratio test and 10-fold cross-validation to determine the optimal regularization parameter λ. A value of λ with a standard error of 1 was chosen to ensure optimal simplicity and predictive effectiveness of the model. The screened variables were then included in a multivariate logistic regression model. The nomogram function in the “rms” package was used to construct a visualization of the effect of each predictor on the outcome variable. The discriminative power of the model was assessed by receiver operating characteristic (ROC) curve analysis performed by the “ROCR” package, and the area under the curve (AUC) value of the model was calculated. The calibration performance of the model was comprehensively evaluated by generating calibration curves using the “rms” package, enabling a detailed comparison between predicted probabilities and actual outcomes. Decision curve analysis was performed using the “rmda” package to comprehensively evaluate the clinical utility of the nomogram, and clinical impact curves were constructed to quantify the potential impact of the model at different clinical thresholds. Additionally, internal validation of the model was achieved through self-sampling to ensure robustness. The internal validation set was used to validate the generalizability and calibration of the model by comparing AUC, calibration curves, decision curve and clinical impact curves.

## 3 Results

### 3.1 Prevalence and baseline profiling

This study included 847 patients aged 41–91 years who were diagnosed with both T2DM and osteoporosis. Among them, 287 patients (age range 41–89 years, mean age 65.49 ± 11.31) were classified in the sarcopenia group, while the remaining 560 patients (age range 41–91 years, mean age 65.33 ± 9.57) were in the non-sarcopenia group. The overall prevalence of sarcopenia in these patients was 33.88% (see **Supplementary Table S1** for baseline data). Due to significant gender differences in the diagnostic criteria for sarcopenia, this study stratified by gender. The prevalence of sarcopenia was significantly higher in male patients compared to female patients (56.84% *vs*. 25.12%, P<0.05). Additionally, male patients with sarcopenia had higher values of age, WWI, neutrophil count, but lower BMI, weight, waist circumference, grip strength, gait speed, lymphocyte count, hemoglobin, albumin, alanine aminotransferase, creatine kinase (CK), uric acid, triglycerides, high-density lipoprotein cholesterol, calcium, phosphorus, 25-Hydroxyvitamin D (25(OH)D), N-mid osteocalcin, and N-terminal propeptide of type I procollagen. In female patients with sarcopenia, higher values were observed for WWI, heart rate, and low-density lipoprotein cholesterol (LDL-C), whereas lower values were noted for BMI, weight, waist circumference, grip strength, gait speed, alanine aminotransferase, CK, creatinine, uric acid, total triglycerides, very low-density lipoprotein cholesterol, phosphorus, as well as lower prevalence rates of hypertension and use of hypoglycemic drugs (see **Supplementary Table S2**).

### 3.2 Clinical screening modeling for male patients

#### 3.2.1 Variable screening results

**Figure 1:**
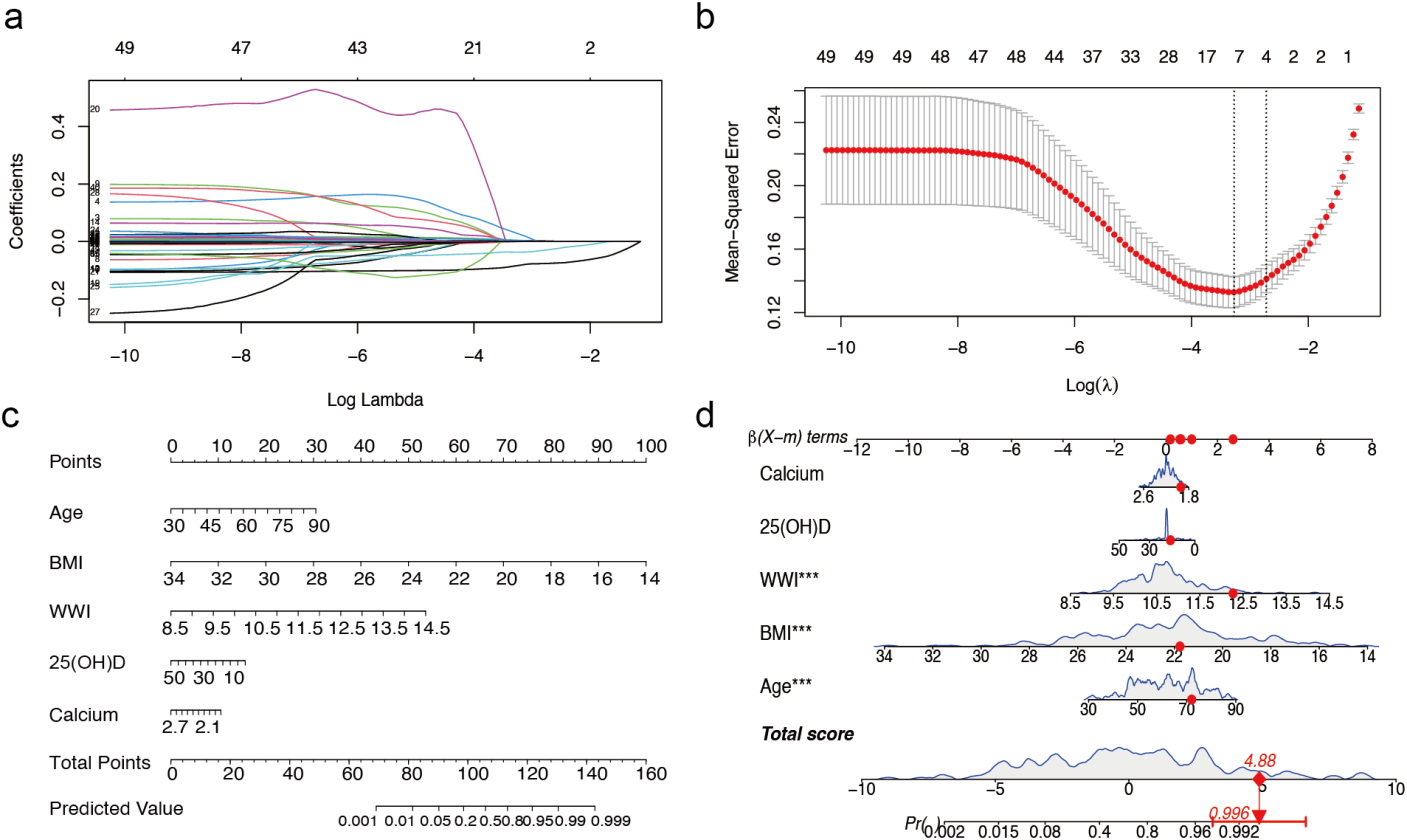
Predictor selection and nomogram for prediction of sarcopenia in males with T2DM combined with osteoporosis. **(a)** LASSO coefficient distributions for 49 risk factors. **(b)** Selection of the minimum criterion (left dashed line) and the 1- standard error criterion (right dashed line) based on the miniature LASSO regression deviation (lambda). **(c)** A nomogram for predicting sarcopenia in males. **(d)** Example of a dynamic nomogram for predicting sarcopenia in a male individual. The significance of the asterisk next to each variable in part D represents the significance of all risk factors. T2DM, type 2 diabetes mellitus; LASSO, least absolute shrinkage and selection operator; BMI, Body Mass Index; WWI, weight-adjusted-waist index; 25(OH)D, 25-Hydroxyvitamin D. *** represents P < 0.001.

Of the 49 parameters collected from male patients, five key characteristics were identified using LASSO logistic regression analysis based on non-zero coefficients (**Figures 1a, b**). These parameters were age, BMI, WWI, 25(OH)D, and calcium, which were subsequently incorporated into the multifactorial logistic regression analysis (**Table 1**).

**Table 1:** Multivariate analysis of predictors selected by the LASSO regression procedure in the male training cohort.

Variables	Estimate	Estimate OR (95% CI)	P
Age	0.0953	6.728 (2.907, 15.568)	<0.0001
BMI	-0.9373	0.037 (0.015, 0.091)	<0.0001
WWI	1.6748	4.525 (2.346, 8.725)	<0.0001
25-Hydroxy Vitamin D	-0.0586	0.928 (0.856, 1.006)	0.0708
Calcium	-2.1894	0.621 (0.331, 1.167)	0.1388

BMI, Body Mass Index; WWI, weight-adjusted-waist index.

#### 3.2.2 Nomogram development

The overall male cohort was randomly divided into a training cohort (70%) and a validation cohort (30%), with baseline data presented in **Supplementary Table S3**. Based on the selected variables, we developed a nomogram model for individualized prediction of sarcopenia risk in male patients (**Figure 1c**). The nomogram consisted of 8 rows, with rows 2–6 corresponding to model variables. Each row’s risk score was determined by the coefficients from the logistic regression model. For example, a 72-year-old male patient with T2DM and osteoporosis, with a BMI of 21.77 kg/m², WWI of 12.27 cm/√kg, calcium 1.94 mmol/L, and 25(OH)D 16.12 ng/ml, had a predicted probability of sarcopenia occurrence of 99.6% according to the model (**Figure 1d**).

#### 3.2.3 Evaluation of model performance

**Figure 2:**
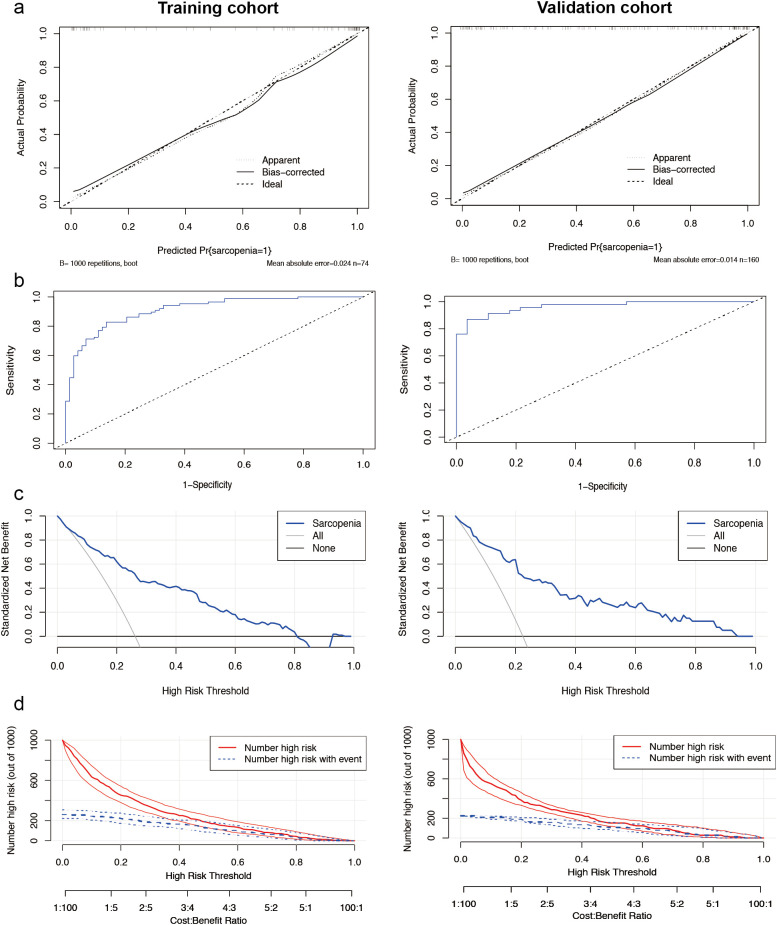
Performance of the male nomogram model. **(a)** Sarcopenia screening model calibration curves. The left side displays the calibration curves for the training cohort, while the right side presents the calibration curves for the internal validation cohort. In these plots, the diagonal dashed line represents the ideal curve, the solid line indicates the bias-corrected curve, and the dotted line shows the actual observed curve. **(b)** The area under the curve was 0.912 (95% CI 0.868-0.955) for the training cohort (left side) and 0.964 (95% CI 0.929-0.999) for the internal validation cohort (right side). Demonstrating the high ability to screen for sarcopenia. **(c)** Analysis of the decision curve for the nomogram assessing sarcopenia risk. Decision curve analysis of the sarcopenia risk nomogram for the training set (left) and the internal validation cohort (right), while Y-axis measures standardized net benefit. The grey line indicates the hypothesis that all patients have sarcopenia and receive treatment, the black line indicates the hypothesis that all patients have no sarcopenia and none receive treatment (net benefit of 0), and the blue line indicates the risk plot. **(d)** Clinical impact curves of the nomogram from the training set (left) and the internal validation cohort (right). Out of 1000 patients, the solid red line indicates the total number of people considered high risk at each risk threshold. The blue dashed line indicates how many of these were true positives (cases).

The calibration curve of the nomogram (**Figure 2a**) demonstrated a high concordance between the predicted probabilities and the actual observations. The ROC curve was employed to assess the discriminatory power of the nomogram, yielding an AUC of 0.912 (95% CI 0.868-0.955) for the training cohort and an AUC of 0.964 (95% CI 0.929-0.999) for the internal validation cohort. In the training cohort, the sensitivity at the maximum Youden index of the ROC curve was 82.8% while the specificity was 86.3%. In the internal validation cohort, the corresponding values were 87.0% and 96.4%, respectively (**Figure 2b**). These results indicate the high predictive accuracy of the model. Furthermore, the decision curve analysis demonstrated robust clinical applicability of the model across various clinical thresholds (**Figure 2c**). The clinical decision curve of the model, depicted in **Figure 2d**, shows that out of 1000 patients, the solid red line represents the total number of individuals considered high risk at each threshold, while the blue dashed line indicates true positive cases.

### 3.3 Clinical screening modeling for female patients

#### 3.3.1 Variable screening results

**Figure 3:**
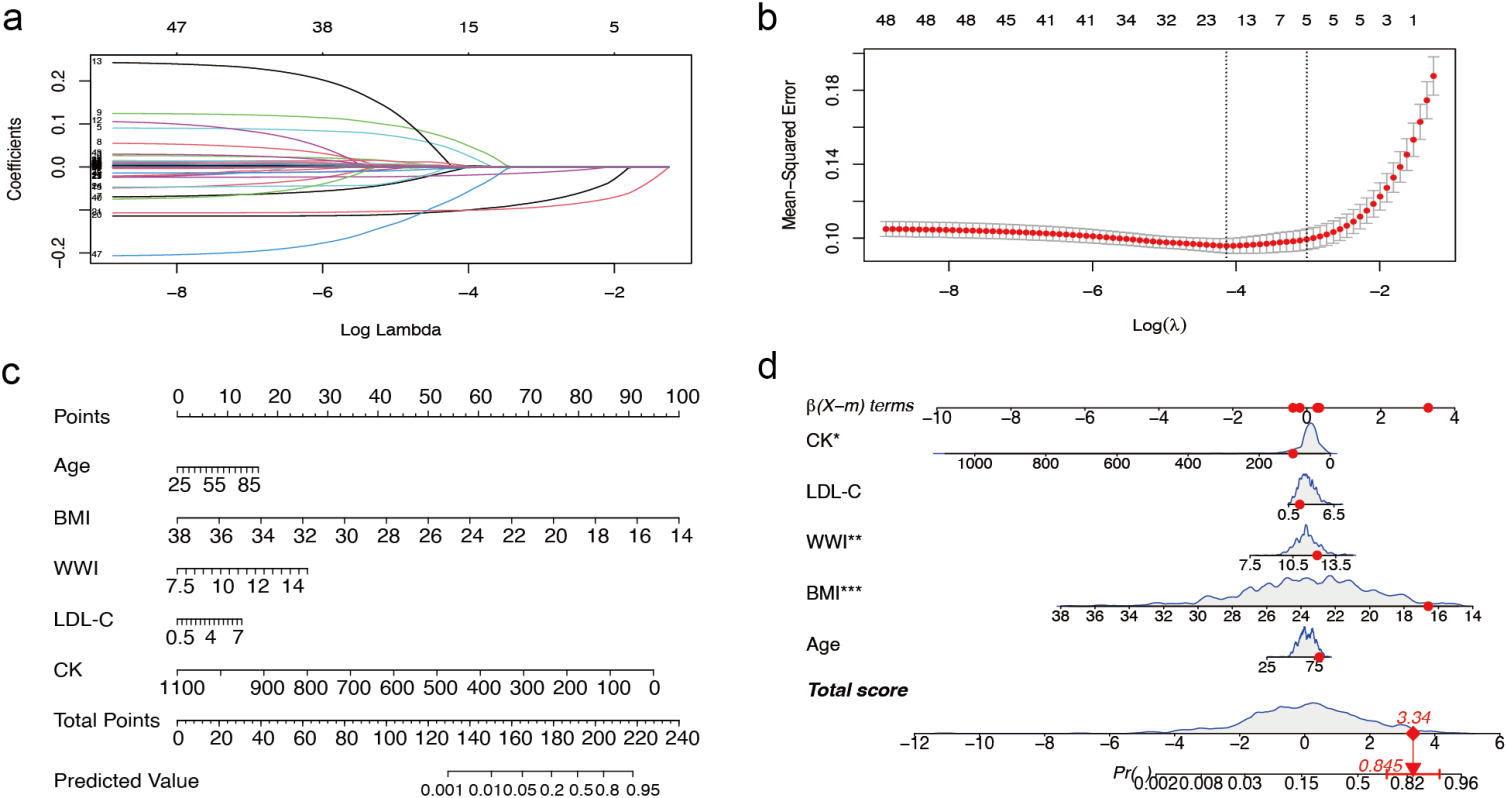
Predictor selection and nomogram construction for sarcopenia in women with T2DM combined with osteoporosis **(a)** LASSO coefficient profiles of the 49 risk factors. **(b)** Selection of the minimum criterion (left dashed line) and the 1- standard error criterion (right dashed line) based on the miniature LASSO regression deviation (lambda). **(c)** A nomogram for predicting sarcopenia in females. First, for each variable for a patient, a point was found on the highest rule, then all points were summed and the total number of points was collected. Finally, find the corresponding predicted probability of sarcopenia on the lowest rule. **(d)** Example of a dynamic nomogram for predicting sarcopenia in a female individual. T2DM, type 2 diabetes mellitus; LASSO, Least Absolute Shrinkage and Selection Operator; BMI, Body Mass Index; WWI, weight-adjusted-waist index; LDL-C, low-density lipoprotein cholesterol; CK, Creatine Kinase. ** represents P < 0.01 and *** represents P < 0.001.

In female patients, the LASSO regression analysis process was similar to that of male patients, as illustrated in **Figures 3a** and **b**, which demonstrate the screening process and coefficient plots of the variables. Five variables were selected: age, BMI, WWI, LDL-C, and CK (**Table 2**), which were subsequently included in the multivariate logistic regression analysis.

**Table 2:** Multivariate analysis of predictors screened by the LASSO regression procedure in the female training cohort.

Variables	Estimate	Estimate OR (95% CI)	*P*
Age	0.0257	1.361 (0.979, 1.893)	0.0666
BMI	-0.4641	0.113 (0.074, 0.171)	<0.0001
WWI	0.3854	1.461 (1.121, 1.903)	0.0050
LDL-C	0.2043	1.350 (0.997, 1.829)	0.0524
Creatine kinase	-0.0096	0.874 (0.784, 0.974)	0.0151

BMI, Body Mass Index; WWI, weight-adjusted-waist index; LDL-C, low-density lipoprotein cholesterol.

#### 3.3.2 Nomogram development

We randomly divided the overall female study sample into a training cohort (70%) and a validation cohort (30%) in a 7:3 ratios, with baseline data shown in **Supplementary Table S4**. The nomogram model developed for female patients (**Figure 3C**) also consisted of 8 rows, where rows 2–6 corresponded to the model variables. For instance, an 80-year-old female patient with T2DM and osteoporosis, with a BMI of 16.59 kg/m², WWI 12.20 cm/√kg, a CK level of 107 mmol/L, and a LDL-C level of 1.97 mmol/L, had a predicted probability of sarcopenia of 84.5% according to the model (**Figure 3D**).

#### 3.3.3 Evaluation of model performance

**Figure 4:**
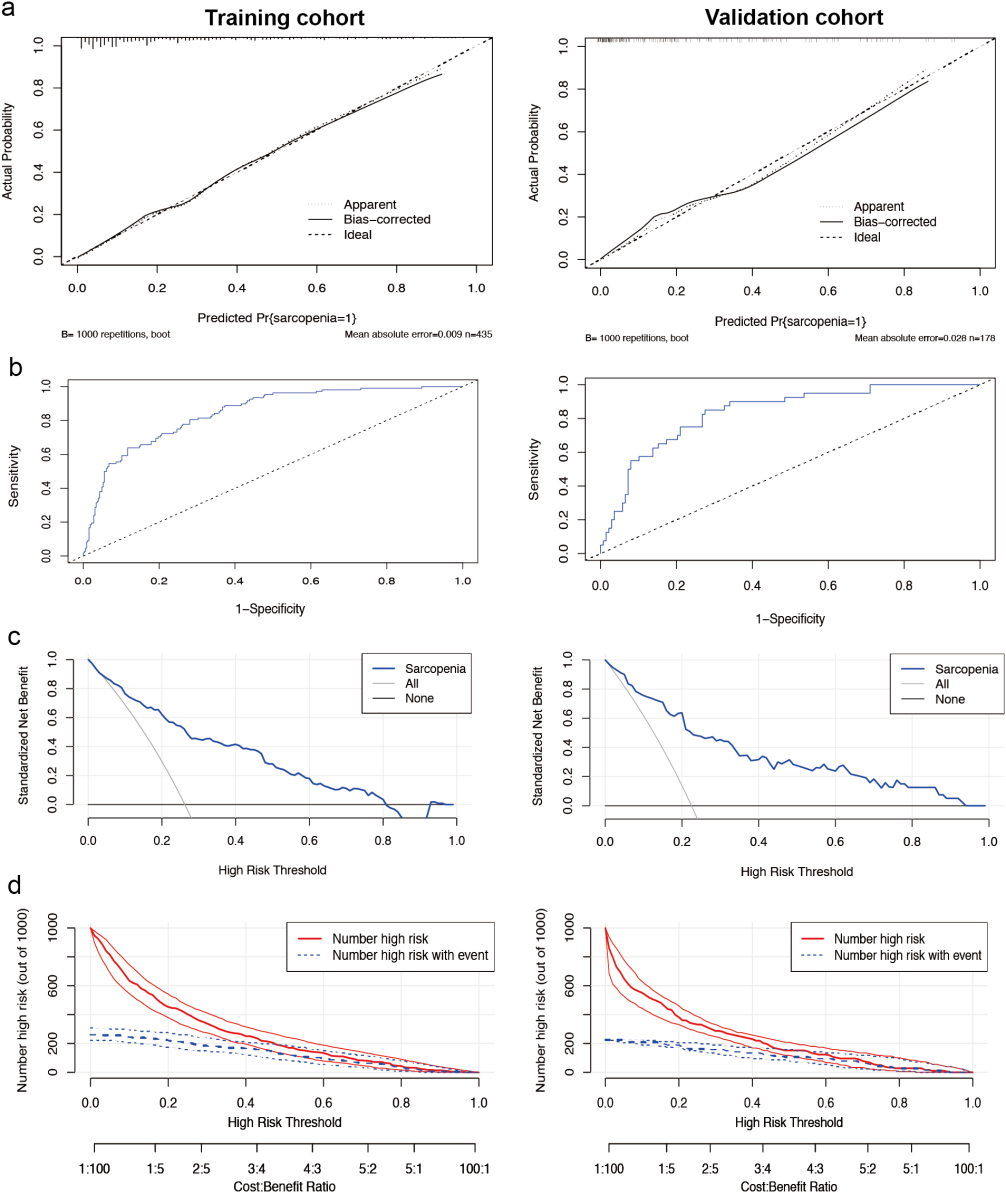
Performance of the nomogram model for females. **(a)** Calibration curves for the training set (left) and internal validation cohort (right) of the female sarcopenia screening model. The diagonal dashed line represents the ideal curve, the solid line indicates the bias-corrected curve, and the dotted line shows the actual observed curve. **(b)** Receiver operating characteristic curves for the training cohort (left side) and the internal validation cohort (right side). The area under the curve was 0.845 (95% CI 0.804-0.885) for the training cohort and 0.836 (95% CI 0.769-0.904) for the internal validation cohort, both of which showed a high ability to screen for sarcopenia. **(c)** Decision curve analysis of the sarcopenia risk nomogram for the training set (left) and the internal validation cohort (right), while Y-axis measures standardized net benefit. The grey line indicates the hypothesis that all patients have sarcopenia and receive treatment, the black line indicates the hypothesis that all patients have no sarcopenia and none receive treatment (net benefit of 0), and the blue line indicates the risk map. **(d)** Clinical impact curves of the nomogram from the training set (left) and the internal validation cohort (right). Out of 1000 patients, the solid red line indicates the total number of people considered high risk at each risk threshold. The blue dashed line indicates how many of these were true positives (cases).

The calibration plots for female patients were in clear agreement with the ideal diagonal (**Figure 4a**). The ROC curves demonstrated high accuracy and discrimination, with an AUC of 0.845 (95% CI: 0.804-0.885) for the training cohort and 0.836 (95% CI: 0.769-0.904) for the internal validation cohort. In the training cohort, the sensitivity at the maximum Youden index of the ROC curve was 86.8%, and the specificity was 67.0%. In the internal validation cohort, the corresponding values were 85.0% and 73.5%, respectively (**Figure 4b**).

#### 3.3.4 Decision curve analysis and model validation

Decision curve analysis (**Figure 4c**) for female patients suggests evidence of high clinical applicability of the model in both the training and validation cohorts. **Figure 4d** presents the clinical decision curves for this model in detail. Of the 1000 patients included in the analysis, the solid red line indicates the total number of patients judged to be at high risk at each risk threshold. Meanwhile, the blue dashed line clearly shows the number of these patients who tested positive.

### 3.4 Correlation analysis of muscle mass

**Figure 5:**
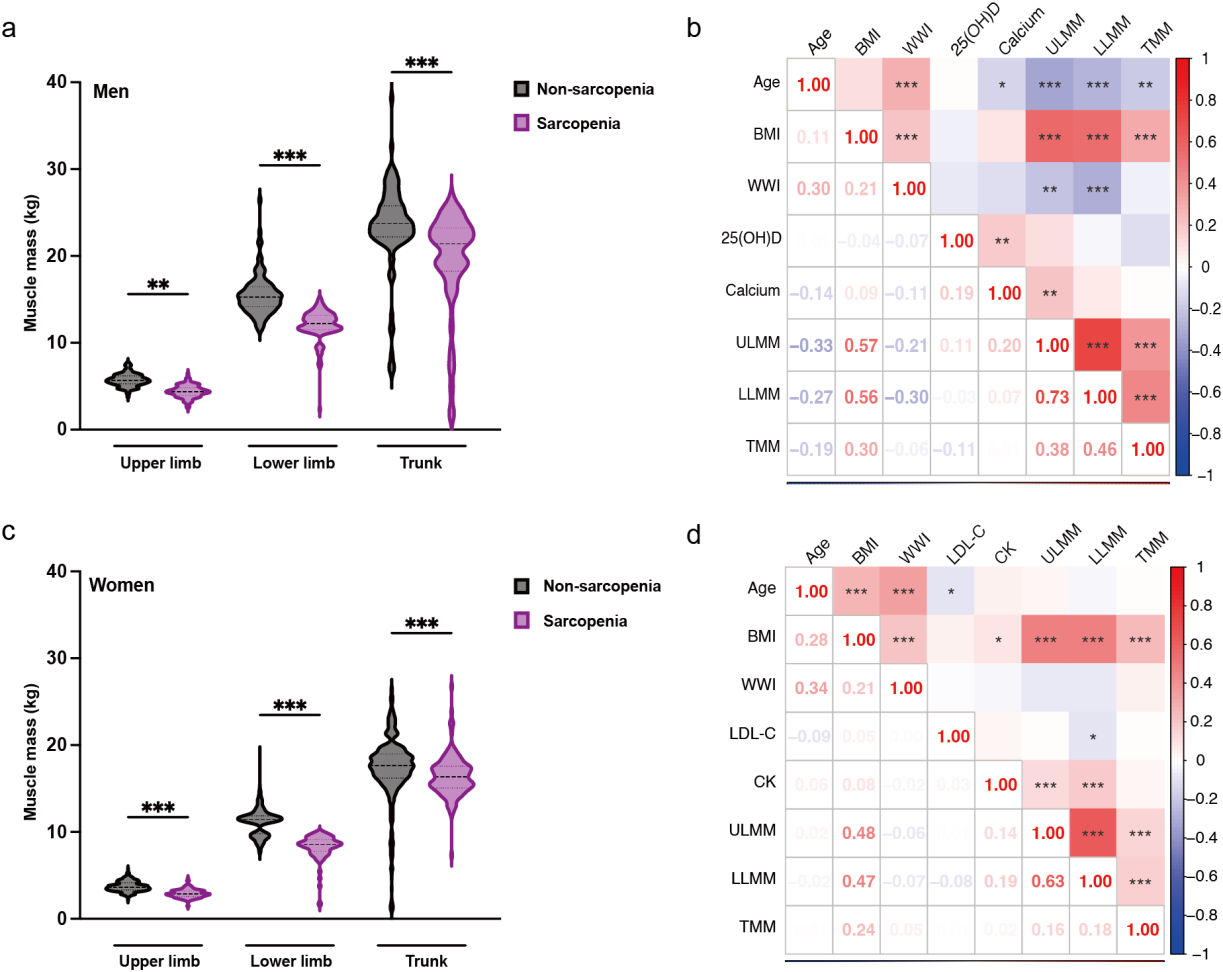
Gender-specific muscle mass changes and correlation analysis. **(a)** Changes in muscle mass by component in males with sarcopenia. **(b)** Heat map of the correlation between each component of muscle and risk factors in males. **(c)** Changes in muscle mass by component in female patients with sarcopenia. **(d)** Heat map of the correlation between each component of muscle and risk factors in females. The results are from the student’s t-test and Spearman’s correlation analysis, * represents P < 0.05, ** represents P < 0.01, and ***represents P < 0.001. ULMM, upper limb muscle mass; LLMM, lower limb muscle mass; TMM, trunk muscle mass; BMI, Body Mass Index; WWI, weight-adjusted-waist index; 25(OH)D, 25-Hydroxyvitamin D; LDL-C, low-density lipoprotein cholesterol; CK, Creatine Kinase.

In male patients, muscle mass of the upper limbs, lower limbs, and trunk significantly decreased in the sarcopenia group (**Figure 5a**). Upper limb muscle mass was negatively correlated with age and WWI, and positively correlated with BMI and serum calcium. Lower limb muscle mass exhibited a significant negative correlation with both age and WWI, and a positive correlation with BMI. Similarly, trunk muscle mass was negatively correlated with age and positively correlated with BMI (**Figure 5b**). In female patients, the muscle mass of the upper limbs, lower limbs, and the trunk was decreased in the sarcopenia group, with the greatest decrease in muscle mass in the lower limbs (**Figure 5c**). Upper limb muscle mass was positively correlated with both BMI and CK. Lower limb muscle mass was significantly negatively correlated with LDL-C and positively correlated with BMI and CK. Additionally, trunk muscle mass was positively correlated with BMI (**Figure 5d**).

## 4 Discussion

In this clinical observation study, it was found that sarcopenia is significantly more prevalent in a population with T2DM combined with osteoporosis, with gender differences. By analyzing gender-specific risk factors, including age, BMI, WWI, vitamin D, calcium, LDL-C, and CK, we could construct an effective predictive model to identify the risk of sarcopenia in patients with T2DM combined with osteoporosis. We anticipate that this model will enhance clinicians’ understanding of early diagnosis and intervention for sarcopenia.

Sarcopenia and osteoporosis often coexist as comorbidities in the elderly, especially in diabetic populations (6). Our findings suggest that the prevalence of sarcopenia is significantly higher in the T2DM combined with osteoporosis population than in the general population and the T2DM-alone population (13), which is consistent with previous studies (14). This finding indicates that sarcopenia comorbidity is more prevalent in patients with T2DM combined with osteoporosis, potentially exacerbating clinical symptoms and complication risks. It also underscores the importance of screening for sarcopenia in this specific population, as early identification and intervention can significantly reduce the risk of falls, fractures, and subsequent disability and death. Additionally, the key findings of the study emphasize the importance of gender in disease development. We found significant differences in the incidence of sarcopenia by gender, with men having a much higher incidence than women, which is consistent with many studies (13, 15, 16). This difference may be related to gender differences in body composition and metabolism, hormone levels, lifestyle habits, disease management, and genetic factors. Males and females differ in body composition distribution and metabolic rate (17), with males tending to have a lower percentage of body fat and a higher basal metabolic rate, which may influence the rate of muscle mass change and muscle loss during aging or stress. Additionally, sex hormones like testosterone significantly affect muscle growth and maintenance. Testosterone levels in men are typically higher than in women, helping to maintain higher muscle mass. As men age, a sharp decline in testosterone levels may lead to a more rapid loss of muscle mass (18). Finally, the later onset of osteoporosis in men is also a contributing factor. Our results suggest that men are more susceptible to sarcopenia in the context of T2DM combined with osteoporosis. Thus, our study highlights the importance of gender-specific strategies in managing diabetes and osteoporosis, particularly the need for early identification and intervention in male patients.

The nomogram was constructed based on an in-depth analysis of patients with T2DM combined with osteoporosis. We first identified several clinical indicators associated with the risk of sarcopenia through LASSO statistical analysis, including age, BMI, WWI, 25(OH)D, calcium, LDL-C, and CK. Subsequently, we constructed a prediction model using these indicators, which was then optimized and validated through ROC curve and decision curve analyses. The predictive efficacy of the nomogram was assessed by the area under the ROC curve (19). In male patients, the AUC reached 91.2%, while in female patients, it was 84.5%. These high AUC values indicate that the nomogram has high sensitivity and specificity, effectively differentiating between sarcopenia and non-sarcopenia patients. The gender-specific design of the nomogram takes into account the physiological and metabolic differences between males and females, which are rarely reported in the existing literature. This design improves the applicability and accuracy of the model for gender-specific patients. Meanwhile, the simplicity of the nomogram makes it easy to use widely in clinical practice, helping to improve the early diagnosis of sarcopenia. Additionally, in our muscle mass correlation analysis, the correlation between these clinical indicators and lower limb muscle mass was significant. This suggests that the nomogram composed of these indicators is more responsive to the loss of muscle mass in the lower limbs and the consequent risk of falls and fractures.

Age is an established factor in the development of sarcopenia, characterized by a gradual decline in muscle mass and function. Studies have shown that in older adults, the rate of muscle protein synthesis decreases while catabolism increases, leading to a reduction in muscle mass and strength (20). In the present study, age was used as an independent predictor of sarcopenia, which is consistent with the existing conclusions. Current research indicates a complex and ambiguous relationship between BMI and sarcopenia. Our study showed a negative association between BMI and sarcopenia, indicating that lower BMI predicts lower muscle mass and a higher risk of sarcopenia in patients with T2DM combined with osteoporosis, which is consistent with some studies. Molly Curtis found that low BMI was significantly associated with a higher likelihood of sarcopenia in community-dwelling older adults in the UK, highlighting that individuals with low BMI are an important risk group for sarcopenia (21). Another 10-year follow-up cohort study in Brazil showed that low BMI was significantly associated with sarcopenia, with lower BMI correlating with higher odds of sarcopenia (22). However, other studies have suggested that higher BMI may mask differences in muscle mass and body fat distribution, leading to the phenomenon known as “Sarcopenic Obesity” (23). The inability of BMI to accurately differentiate between muscle mass and body fat proportions may be one reason for the discrepancy in conclusions (24). WWI is a new type of obesity assessment index that combines the advantages of waist circumference in reflecting abdominal obesity, while reducing the correlation with BMI by adjusting for body weight. WWI is superior to BMI and waist circumference in assessing muscle and fat mass, and shows a positive correlation with total abdominal fat area and a negative correlation with total abdominal muscle area (25, 26). Recent studies have shown that for every 1-unit increase in WWI, the risk of sarcopenia increases 14.55-fold in men and 2.86-fold in women, suggesting an increased risk of sarcopenia in individuals with higher WWI (27), which is consistent with our results.

In the male group, we found that reductions in both vitamin D and calcium were associated with the risk of sarcopenia. The relationship between vitamin D and sarcopenia has been widely studied and observed. According to a recent study, there is a significant non-linear association between decreased vitamin D levels and an increased risk of sarcopenia. Particularly, at serum 25(OH)D levels below 20 ng/mL, the risk of sarcopenia increases dramatically with decreasing vitamin D levels (28). Another study also found that vitamin D deficiency was significantly associated with reduced muscle strength, and that vitamin D can directly promote muscle fiber proliferation and differentiation by binding to vitamin D receptors in skeletal muscle cells (29). Additionally, a cross-sectional analysis using the UK Biobank showed that higher calcium intake was associated with lower odds of sarcopenia (30). Another analysis of the Korean National Health and Nutrition Examination Survey found that daily calcium intake was positively associated with lower limb skeletal muscle mass (31). Reduced calcium levels may affect muscle contraction and nerve conduction, further impacting muscle strength and function, which is consistent with the features of sarcopenia (32). Therefore, our study supports the importance of maintaining adequate vitamin D and calcium levels for the prevention and treatment of sarcopenia.

Meanwhile, our study demonstrated that elevated LDL-C levels increase the risk of sarcopenia in the female population. Several studies have indicated that the increase in LDL-C, a parameter related to lipid metabolism, is a risk factor for sarcopenia (33). Another study revealed a negative correlation between LDL-C levels and skeletal muscle mass, emphasizing the potential role of abnormal lipid metabolism in the development of sarcopenia (34). However, the relationship between lipid levels and sarcopenia may be influenced by various factors, including diet, lifestyle, and other metabolic factors, necessitating further studies to elucidate the exact mechanisms linking lipid metabolism and sarcopenia. The correlation between CK and sarcopenia in females was an interesting finding in this study, and we found that reduced CK was an independent predictor of sarcopenia development. A cross-sectional study on the relationship between serum CK and sarcopenia showed that CK was positively correlated with skeletal muscle mass in patients with T2DM, which is in agreement with our results, suggesting that CK may be used as a biomarker to assess the risk of sarcopenia in patients with T2DM.

This study has several limitations. First, the sample size, as well as regional and ethnic differences, may affect the generalizability of the results. Second, due to the cross-sectional design of the study, it was not possible to establish a causal relationship between these risk factors and sarcopenia. Finally, our study did not consider other factors that may influence sarcopenia, such as genetic factors, lifestyle habits, nutritional status, and environmental factors. Future studies could improve the representativeness and generalizability of the findings by expanding the sample size and including genetic, lifestyle and environmental factors. Follow-up studies can also be conducted to assess the long-term predictive ability of the nomogram in practical clinical applications.

In summary, this study provides a scientific basis for the early identification and intervention of sarcopenia in patients with T2DM combined with osteoporosis by establishing a gender-specific nomogram. It also highlights the need for gender-specific strategies in disease management. Despite the limitations of sample representativeness and cross-sectional design, this study provides an accurate, low-economic cost, non-invasive screening tool for early identification of people at high risk of fractures and falls. Further long-term prospective studies are necessary to validate the predictive power and clinical effectiveness of the nomogram.

## Data Availability

The raw data supporting the conclusions of this article will be made available by the authors, without undue reservation.

## References

[1] Sun H Saeedi P Karuranga S Pinkepank M Ogurtsova K Duncan BB. IDF Diabetes Atlas: Global, regional and country-level diabetes prevalence estimates for 2021 and projections for 2045. Diabetes Res Clin Pract. (2022) 183:109119. doi: 10.1016/j.diabres.2021.109119 34879977 PMC11057359

[2] Yuan S Larsson SC. Epidemiology of sarcopenia: Prevalence, risk factors, and consequences. Metabolism. (2023) 144:155533. doi: 10.1016/j.metabol.2023.155533 36907247

[3] Xiao PL Cui AY Hsu CJ Peng R Jiang N Xu XH. Global, regional prevalence, and risk factors of osteoporosis according to the World Health Organization diagnostic criteria: a systematic review and meta-analysis. Osteoporos Int. (2022) 33:2137–53. doi: 10.1007/s00198-022-06454-3 35687123

[4] Cipriani C Colangelo L Santori R Renella M Mastrantonio M Minisola S. The interplay between bone and glucose metabolism. Front Endocrinol (Lausanne). (2020) 11:122. doi: 10.3389/fendo.2020.00122 32265831 PMC7105593

[5] Salom Vendrell C García Tercero E Moro Hernández JB Cedeno-Veloz BA. Sarcopenia as a little-recognized comorbidity of type II diabetes mellitus: A review of the diagnosis and treatment. Nutrients. (2023) 15:4149. doi: 10.3390/nu15194149 37836433 PMC10574035

[6] Polito A Barnaba L Ciarapica D Azzini E. Osteosarcopenia: A narrative review on clinical studies. Int J Mol Sci. (2022) 23:5591. doi: 10.3390/ijms23105591 35628399 PMC9147376

[7] Huo YR Suriyaarachchi P Gomez F Curcio CL Boersma D Muir SW. Phenotype of osteosarcopenia in older individuals with a history of falling. J Am Med Dir Assoc. (2015) 16:290–5. doi: 10.1016/j.jamda.2014.10.018 25512216

[8] Locquet M Beaudart C Bruyère O Kanis JA Delandsheere L Reginster JY. Bone health assessment in older people with or without muscle health impairment. Osteoporos Int. (2018) 29:1057–67. doi: 10.1007/s00198-018-4384-1 PMC5948285 29445830

[9] Gielen E Dupont J Dejaeger M Laurent MR. Sarcopenia, osteoporosis and frailty. Metabolism. (2023) 145:155638. doi: 10.1016/j.metabol.2023.155638 37348597

[10] Fang F Feng B Gao Y Gong Q Gong Y Gu Z. Clinical guidelines for prevention and treatment of type 2 diabetes mellitus in the elderly in China (2022 edition). Zhonghua Nei Ke Za Zhi. (2022) 61:12–50. doi: 10.3760/cma.j.cn112138-20211027-00751 34979769

[11] Kanis JA. Assessment of fracture risk and its application to screening for postmenopausal osteoporosis: synopsis of a WHO report. WHO Study Group Osteoporos Int. (1994) 4:368–81. doi: 10.1007/BF01622200 7696835

[12] Chen LK Woo J Assantachai P Auyeung TW Chou MY Iijima K. Asian working group for sarcopenia: 2019 consensus update on sarcopenia diagnosis and treatment. J Am Med Dir Assoc. (2020) 21:300–7.e2. doi: 10.1016/j.jamda.2019.12.012 32033882

[13] Ai Y Xu R Liu L. The prevalence and risk factors of sarcopenia in patients with type 2 diabetes mellitus: a systematic review and meta-analysis. Diabetol Metab Syndr. (2021) 13:93. doi: 10.1186/s13098-021-00707-7 34479652 PMC8414692

[14] Gao Q Hu K Yan C Zhao B Mei F Chen F. Associated factors of sarcopenia in community-dwelling older adults: A systematic review and meta-analysis. Nutrients. (2021) 13:4291. doi: 10.3390/nu13124291 34959843 PMC8707132

[15] Yu M Pan M Liang Y Li X Li J Luo L. A nomogram for screening sarcopenia in Chinese type 2 diabetes mellitus patients. Exp Gerontol. (2023) 172:112069. doi: 10.1016/j.exger.2022.112069 36535452

[16] Sazlina SG Lee PY Chan YM MS AH Tan NC. The prevalence and factors associated with sarcopenia among community living elderly with type 2 diabetes mellitus in primary care clinics in Malaysia. PloS One. (2020) 15:e0233299. doi: 10.1371/journal.pone.0233299 32433712 PMC7239480

[17] Bellissimo MP Fleischer CC Reiter DA Goss AM Zhou L Smith MR. Sex differences in the relationships between body composition, fat distribution, and mitochondrial energy metabolism: a pilot study. Nutr Metab (Lond). (2022) 19:37. doi: 10.1186/s12986-022-00670-8 35597962 PMC9123728

[18] Ketchem JM Bowman EJ Isales CM. Male sex hormones, aging, and inflammation. Biogerontology. (2023) 24:1–25. doi: 10.1007/s10522-022-10002-1 36596999 PMC9810526

[19] Park SY. Nomogram: An analogue tool to deliver digital knowledge. J Thorac Cardiovasc Surg. (2018) 155:1793. doi: 10.1016/j.jtcvs.2017.12.107 29370910

[20] Larsson L Degens H Li M Salviati L Lee YI Thompson W. Sarcopenia: aging-related loss of muscle mass and function. Physiol Rev. (2019) 99:427–511. doi: 10.1152/physrev.00061.2017 30427277 PMC6442923

[21] Curtis M Swan L Fox R Warters A O'Sullivan M. Associations between body mass index and probable sarcopenia in community-dwelling older adults. Nutrients. (2023) 15:1505. doi: 10.3390/nu15061505 36986233 PMC10059806

[22] Pereira CC Pagotto V de Oliveira C Silveira EA. Sarcopenia and mortality risk in community-dwelling Brazilian older adults. Sci Rep. (2022) 12:17531. doi: 10.1038/s41598-022-22153-9 36266412 PMC9585028

[23] Bilski J Pierzchalski P Szczepanik M Bonior J Zoladz JA. Multifactorial mechanism of sarcopenia and sarcopenic obesity. Role of physical exercise, microbiota and myokines. Cells. (2022) 11:160. 35011721 10.3390/cells11010160 PMC8750433

[24] Merchant RA Seetharaman S Au L Wong MWK Wong BLL Tan LF. Relationship of fat mass index and fat free mass index with body mass index and association with function, cognition and sarcopenia in pre-frail older adults. Front Endocrinol (Lausanne). (2021) 12:765415. doi: 10.3389/fendo.2021.765415 35002957 PMC8741276

[25] Kim KJ Son S Kim KJ Kim SG Kim NH. Weight-adjusted waist as an integrated index for fat, muscle and bone health in adults. J Cachexia Sarcopenia Muscle. (2023) 14:2196–203. doi: 10.1002/jcsm.13302 PMC10570086 37550773

[26] Kim JY Choi J Vella CA Criqui MH Allison MA Kim NH. Associations between weight-adjusted waist index and abdominal fat and muscle mass: multi-ethnic study of atherosclerosis. Diabetes Metab J. (2022) 46:747–55. doi: 10.4093/dmj.2021.0294 PMC9532169 35350091

[27] Zhou H Su H Gong Y Chen L Xu L Chen G. The association between weight-adjusted-waist index and sarcopenia in adults: a population-based study. Sci Rep. (2024) 14:10943. doi: 10.1038/s41598-024-61928-0 38740910 PMC11091224

[28] Sha T Wang Y Zhang Y Lane NE Li C Wei J. Genetic variants, serum 25-hydroxyvitamin D levels, and sarcopenia: A mendelian randomization analysis. JAMA Netw Open. (2023) 6:e2331558. doi: 10.1001/jamanetworkopen.2023.31558 37647062 PMC10469287

[29] Mizuno T Hosoyama T Tomida M Yamamoto Y Nakamichi Y Kato S. Influence of vitamin D on sarcopenia pathophysiology: A longitudinal study in humans and basic research in knockout mice. J Cachexia Sarcopenia Muscle. (2022) 13:2961–73. doi: 10.1002/jcsm.13102 PMC9745482 36237134

[30] Petermann-Rocha F Chen M Gray SR Ho FK Pell JP Celis-Morales C. Factors associated with sarcopenia: A cross-sectional analysis using UK Biobank. Maturitas. (2020) 133:60–7. doi: 10.1016/j.maturitas.2020.01.004 32005425

[31] Seo MH Kim MK Park SE Rhee EJ Park CY Lee WY. The association between daily calcium intake and sarcopenia in older, non-obese Korean adults: the fourth Korea National Health and Nutrition Examination Survey (KNHANES IV) 2009. Endocr J. (2013) 60:679–86. doi: 10.1507/endocrj.EJ12-0395 23357977

[32] Ganapathy A Nieves JW. Nutrition and sarcopenia-what do we know? Nutrients. (2020) 12:1755. doi: 10.3390/nu12061755 32545408 PMC7353446

[33] Jiang Y Xu B Zhang K Zhu W Lian X Xu Y. The association of lipid metabolism and sarcopenia among older patients: a cross-sectional study. Sci Rep. (2023) 13:17538. doi: 10.1038/s41598-023-44704-4 37845303 PMC10579328

[34] Gong H Liu Y Lyu X Dong L Zhang X. Lipoprotein subfractions in patients with sarcopenia and their relevance to skeletal muscle mass and function. Exp Gerontol. (2022) 159:111668. doi: 10.1016/j.exger.2021.111668 34954281

