# Supplementary material for: Early identification of sarcopenia in patients with diabetes mellitus combined with osteoporosis: development and validation of a gender-specific nomogram
Source: Front Endocrinol (Lausanne). 2025 Apr 30;16:1590247. doi: 10.3389/fendo.2025.1590247 (PMC12074909; doi:10.3389/fendo.2025.1590247)
Supplement: Supplementary file 1 [file Table1.docx]

Supplementary Material

# Supplementary Tables

**Table S1 Baseline characteristics**

| Variables | non - Sarcopenia  (n= 560) | Sarcopenia  (n= 287 ) | *P* |
| --- | --- | --- | --- |
| Age (year) | 65.33 ± 9.57 | 65.49 ± 11.31 | 0.831 |
| Duration of Diabetes (year) | 8.00 (3.00 - 12.00) | 6.00 (2.00 - 11.00) | 0.038 |
| Use of hypoglycaemic drugs, n (%) | 490 (87.50) | 233 (81.20) | 0.014 |
| Smoking status, n (%) | 43 (7.70) | 56 (19.50) | <0.001 |
| Drinking status, n (%) | 14 (2.50) | 23 (8.00) | <0.001 |
| Hypertension, n (%) | 308 (55.00) | 126 (43.90) | <0.001 |
| ASMI (kg/m^2^) | 6.67 ± 0.86 | 5.47 ± 0.92 | <0.001 |
| Handgrip strength (kg) | 26.25 ± 1.91 | 22.00 ± 1.35 | 0.025 |
| Gait speed (m/s) | 1.19 ± 0.33 | 0.88 ± 0.08 | 0.037 |
| Height (cm) | 154.55 ± 7.59 | 156.83 ± 8.42 | <0.001 |
| Weight (cm) | 58.76 ± 9.15 | 51.24 ± 8.32 | <0.001 |
| BMI (kg/m^2^) | 24.58 ± 3.32 | 20.79 ± 2.69 | <0.001 |
| Waist Circumference (cm) | 86.12 ± 9.63 | 80.15 ± 8.95 | <0.001 |
| WWI (cm/√kg) | 11.26 ± 0.90 | 11.27 ± 0.94 | 0.844 |
| Duration of Hypertension (year) | 1.00 (0.00 - 3.00) | 0.00 (0.00 - 1.00) | 0.002 |
| Systolic Blood Pressure (mmHg) | 139.78 ± 20.61 | 138.08 ± 21.70 | 0.264 |
| Diastolic Blood Pressure (mmHg) | 77.66 ± 10.74 | 76.93 ± 11.17 | 0.352 |
| Heart Rate (beats/min) | 79.21 ± 12.08 | 82.41 ± 12.78 | <0.001 |
| FPG (mmol/l) | 7.67 (5.68 - 10.75) | 7.61 (5.29 - 11.43) | 0.729 |
| HbA1c (%) | 9.13 ± 2.49 | 9.68 ± 2.64 | 0.004 |
| White Blood Cell (×10^9^/L） | 6.50 (5.22 - 7.96) | 6.57 (5.24 - 8.46) | 0.339 |
| Neutrophil (×10^9^/L） | 3.81 (2.92 - 5.12) | 4.15 (2.99 - 5.99) | <0.001 |
| Lymphocyte (×10^9^/L） | 1.89 ± 0.74 | 1.72 ± 0.74 | 0.001 |
| Hemoglobin (g/l) | 125.32 ± 17.86 | 123.37 ± 20.66 | 0.181 |
| Total Bilirubin (μmol/l) | 9.92 ± 4.80 | 10.56 ± 5.81 | 0.206 |
| Direct Bilirubin (μmol/l) | 2.90 (2.20 - 4.00) | 3.30 (2.20 - 4.80) | 0.022 |
| Indirect Bilirubin (μmol/l) | 5.90 (4.30 - 8.20) | 5.80 (4.10 - 8.18) | 0.607 |
| Total Protein (g/l) | 66.67 ± 6.76 | 65.52 ± 7.56 | 0.076 |
| Albumin (g/l) | 38.61 ± 4.97 | 37.44 ± 4.55 | 0.001 |
| Globulin (g/l) | 27.95 ± 4.51 | 28.4 ± 6.66 | 0.423 |
| Alanine Aminotransferase (U/l) | 19.00 (14.00 - 28.00) | 18.00 (13.00 - 27.50) | 0.298 |
| Aspartate Aminotransferase (U/l) | 21.00 (16.00 - 27.00) | 20.00 (16.00 - 26.00) | 0.307 |
| Lactate Dehydrogenase (U/l) | 184.50 (159.25 - 210.00) | 180.00 (156.00 - 219.00) | 0.975 |
| CK (U/l) | 62.50 (45.00 - 92.00) | 51.00 (34.00 - 77.25) | <0.001 |
| Urea (mmol/l) | 5.30 (4.20 - 6.65) | 5.07 (4.00 - 6.60) | 0.340 |
| Creatinine (μmol/l) | 56.00 (46.30 - 69.10) | 56.30 (46.00 - 73.05) | 0.760 |
| Uric Acid (μmol/l) | 290.05 (230.18 - 357.45) | 269 (209.7 - 338.23) | 0.017 |
| TG (mmol/l) | 1.54 (1.01 - 2.28) | 1.17 (0.86 - 1.95) | <0.001 |
| TC (mmol/l) | 4.84 ± 1.34 | 4.67 ± 1.56 | 0.105 |
| HDL - C (mmol/l) | 1.14 (0.94 - 1.42) | 1.17 (0.92 - 1.44) | 0.995 |
| LDL - C (mmol/l) | 2.88 ± 1.02 | 3.05 ± 1.12 | 0.718 |
| VLDL - C (mmol/l) | 0.56 (0.37 - 0.87) | 0.47 (0.34 - 0.67) | <0.001 |
| Calcium (mmol/l) | 2.23 ± 0.15 | 2.2 ± 0.15 | 0.005 |
| Sodium (mmol/l) | 141.54 ± 2.99 | 140.88 ± 3.58 | 0.009 |
| Potassium (mmol/l) | 4.04 ± 0.51 | 3.96 ± 0.54 | 0.043 |
| Phosphorus (mmol/l) | 1.17 ± 0.22 | 1.09 ± 0.22 | <0.001 |
| 25 (OH)D (ng/ml) | 17.31 (13.05 - 22.99) | 14.63 (10.78 - 21.46) | 0.98 |
| N-Mid Osteocalcin (ng/ml) | 44.00 (32.57 - 59.84) | 38.34 (26.11 - 55.17) | 0.005 |
| N-Terminal Propeptide of Type I Collagen (ng/ml) | 0.54 (0.36 - 0.70) | 0.54 (0.36 - 0.75) | 0.015 |
| Beta-C cross-linking peptide (ng/ml) | 62.50 (45.00 - 92.00) | 51.00 (34.00 - 77.25) | 0.834 |

Abbreviations: BMI: Body Mass Index, WWI: weight-adjusted-waist index, FPG: fasting blood glucose, HbA1c: glycosylated hemoglobin, CK: Creatine Kinase, TC: total cholesterol, TG: total triglyceride, HDL-C: high-density lipoprotein cholesterol, LDL-C: low-density lipoprotein cholesterol, VLDL-C: very-low-density lipoprotein cholesterol. 25 (OH)D: 25-Hydroxy Vitamin D.

**Table S2 Baseline characteristics of the population after gender stratification**

| Variables | Men (n= 234) | | | Women (n=613) | | |
| --- | --- | --- | --- | --- | --- | --- |
|  | non-  Sarcopenia ( n =101) | Sarcopenia ( n =133) | *P* | non-  Sarcopenia ( n = 459) | Sarcopenia ( n =154) | *P* |
| Age (year) | 57.53 ± 12.05 | 64.61 ± 12.31 | <0.001 | 67.04 ± 7.99 | 66.26 ± 10.34 | 0.392 |
| Duration of Diabetes (year) | 7.00 (3.00 - 10.00) | 6.00 (2.00 - 10.00) | 0.658 | 10.00 (4.00 - 13.00) | 6.50 (2.00 - 11.00) | 0.053 |
| Use of hypoglycaemic drugs, n (%) | 88 (87.13) | 112 (84.21) | 0.530 | 402 (87.58) | 121 (78.57) | 0.006 |
| Smoking status, n (%) | 40 (39.60) | 55 (41.35) | 0.787 | 3 (0.65) | 1 (0.65) | 0.995 |
| Drinking status, n (%) | 14 (13.86) | 23 (17.29) | 0.476 | 0 (0.00) | 0 (0.00) | NA |
| Hypertension, n (%) | 42 (41.58) | 55 (41.35) | 0.972 | 266 (57.95) | 71 (46.10) | 0.011 |
| Duration of Hypertension (year) | 0.00 (0.00 - 1.00) | 0.00 (0.00 - 1.00) | 0.981 | 1.00 (0.00 - 4.00) | 0.00 (0.00 - 1.00) | 0.009 |
| ASMI (kg/m^2^) | 7.85 ± 0.78 | 6.19 ± 0.67 | <0.001 | 6.41 ± 0.64 | 4.86 ± 0.60 | <0.001 |
| Handgrip strength (kg) | 26.76 ± 1.59 | 23.62 ± 1.56 | 0.020 | 17.85 ± 1.12 | 15.20 ± 0.89 | 0.004 |
| Gait speed (m/s) | 0.93 ± 0.10 | 0.77 ± 0.09 | 0.046 | 1.05 ± 0.09 | 0.78 ± 0.12 | <0.001 |
| Height (cm) | 164.30 ± 6.78 | 163.07 ± 6.58 | 0.163 | 152.40 ± 5.89 | 151.44 ± 5.67 | 0.078 |
| Weight (cm) | 65.60 ± 8.83 | 55.52 ± 7.69 | <0.001 | 57.25 ± 8.51 | 47.54 ± 6.98 | <0.001 |
| BMI (kg/m^2^) | 24.27 ± 2.69 | 20.87 ± 2.59 | <0.001 | 24.65 ± 3.45 | 20.72 ± 2.78 | <0.001 |
| Waist Circumference (cm) | 84.93 ± 8.21 | 80.95 ± 9.16 | 0.001 | 86.39 ± 9.92 | 79.47 ± 8.75 | <0.001 |
| WWI (cm/√kg) | 10.50 ± 0.62 | 10.91 ± 0.86 | <0.001 | 11.43 ± 0.86 | 11.59 ± 0.89 | 0.045 |
| Systolic Blood Pressure (mmHg) | 135.01 ± 18.26 | 135.71 ± 21.37 | 0.793 | 140.83 ± 20.97 | 140.13 ± 21.85 | 0.722 |
| Diastolic Blood Pressure (mmHg) | 80.26 ± 10.59 | 77.8 ± 11.28 | 0.092 | 77.09 ± 10.70 | 76.17 ± 11.06 | 0.359 |
| Heart Rate (beats/min) | 79.65 ± 11.18 | 81.72 ± 12.90 | 0.203 | 79.12 ± 12.28 | 83 ± 12.69 | 0.001 |
| FPG (mmol/l) | 8.04 (5.54 - 12.48) | 7.74 (5.39 - 12.11) | 0.537 | 7.64 (5.69 - 10.44) | 7.46 (5.24 - 11.27) | 0.537 |
| HbA1c (%) | 9.80 ± 2.62 | 10.02 ± 2.78 | 0.543 | 8.99 ± 2.44 | 9.38 ± 2.48 | 0.093 |
| White Blood Cell (×10^9^/L） | 6.41 (5.51 - 7.72) | 7 (5.27 - 8.60) | 0.215 | 6.52 (5.19 - 8.06) | 6.40 (5.17 - 8.28) | 0.980 |
| Neutrophil (×10^9^/L） | 3.71 (3.00 - 4.64) | 4.41 (3.17 - 6.06) | 0.011 | 3.81 (2.91 - 5.2) | 3.79 (2.74 - 5.53) | 0.774 |
| Lymphocyte (×10^9^/L） | 1.87 ± 0.75 | 1.63 ± 0.78 | 0.023 | 1.89 ± 0.74 | 1.79 ± 0.69 | 0.117 |
| Hemoglobin (g/l) | 135.19 ± 19.83 | 127.18 ± 23.19 | 0.006 | 123.18 ± 16.67 | 120.08 ± 17.61 | 0.052 |
| Total Bilirubin (μmol/l) | 10.23 ± 3.97 | 10.47 ± 5.57 | 0.775 | 9.87 ± 4.92 | 10.65 ± 6.06 | 0.211 |
| Direct Bilirubin (μmol/l) | 3.35 (2.70 - 4.20) | 3.30 (2.28 - 5.30) | 0.719 | 2.85 (2.20 - 3.93) | 3.15 (2.20 - 4.30) | 0.125 |
| Indirect Bilirubin (μmol/l) | 6.50 (5.03 - 8.08) | 5.50 (3.60 - 8.60) | 0.264 | 5.80 (4.20 - 8.33) | 5.80 (4.40 - 8.13) | 0.845 |
| Total Protein (g/l) | 65.04 ± 7.69 | 64.22 ± 7.37 | 0.545 | 66.94 ± 6.57 | 66.73 ± 7.58 | 0.795 |
| Albumin (g/l) | 38.70 ± 5.66 | 36.96 ± 4.85 | 0.014 | 38.59 ± 4.81 | 37.83 ± 4.25 | 0.085 |
| Globulin (g/l) | 26.78 ± 4.63 | 27.65 ± 6.69 | 0.428 | 28.14 ± 4.47 | 29.10 ± 6.59 | 0.113 |
| Alanine Aminotransferase (U/l) | 24.00 (17.00 - 43.00) | 19.00 (14.00 - 32.50) | 0.025 | 18.00 (13.00 - 26.00) | 16.00 (11.25 - 25.00) | 0.025 |
| Aspartate Aminotransferase (U/l) | 22.00 (17.00 - 34.00) | 20.00 (16.00 - 28.00) | 0.141 | 20.00 (16.00 - 26.00) | 19.00 (15.25 - 24.75) | 0.141 |
| Lactate Dehydrogenase (U/l) | 176.00 (156.25 - 204.00) | 182.00 (156.00 - 221.00) | 0.247 | 187.00 (160.25 - 210.75) | 177.50 (154.25 - 217.50) | 0.247 |
| CK (U/l) | 83.00 (51.00 - 122.00) | 56.50 (41.75 - 85.25) | 0.013 | 61.00 (45.00 - 88.00) | 43.00 (29.25 - 64.75) | <0.001 |
| Urea (mmol/l) | 5.46 (4.44 - 6.97) | 5.38 (4.05 - 6.96) | 0.566 | 5.29 (4.10 - 6.55) | 4.72 (3.90 - 6.33) | 0.566 |
| Creatinine (μmol/l) | 65.60 (55.40 - 77.20) | 80.50 (65.10 - 98.80) | 0.928 | 53.25 (45.18 - 67.00) | 50.40 (42.00 - 61.90) | 0.001 |
| Uric Acid (μmol/l) | 315.00 (255.10 - 379.00) | 287.00 (231.90 - 351.90) | 0.026 | 282.90 (221.35 - 354.50) | 258.50 (197.60 - 328.10) | 0.026 |
| TG (mmol/l) | 1.24 (0.82 - 2.2) | 1.07 (0.79 - 1.54) | 0.071 | 1.58 (1.05 - 2.31) | 1.31 (0.92 - 1.98) | 0.011 |
| TC (mmol/l) | 4.76 ± 1.59 | 4.46 ± 1.70 | 0.175 | 4.86 ± 1.28 | 4.84 ± 1.41 | 0.933 |
| HDL-C (mmol/l) | 1.13 (0.89 - 1.45) | 1.11 (0.89 - 1.38) | 0.258 | 1.14 (0.96 - 1.42) | 1.21 (0.95 - 1.48) | 0.365 |
| LDL-C (mmol/l) | 2.87 ± 1.00 | 2.72 ± 1.09 | 0.270 | 2.88 ± 1.03 | 3.27 ± 1.04 | 0.039 |
| VLDL - C (mmol/l) | 0.46 (0.30 - 0.65) | 0.42 (0.34-0.60) | 0.747 | 0.59 (0.40 - 0.90) | 0.51 (0.35 - 0.77) | 0.004 |
| Calcium (mmol/l) | 2.24 ± 0.16 | 2.18 ± 0.14 | 0.004 | 2.23 ± 0.15 | 2.22 ± 0.14 | 0.567 |
| Sodium (mmol/l) | 140.93 ± 3.02 | 140.38 ± 3.63 | 0.228 | 141.67 ± 2.97 | 141.31 ± 3.49 | 0.222 |
| Potassium (mmol/l) | 4.05 ± 0.49 | 3.98 ± 0.56 | 0.332 | 4.03 ± 0.51 | 3.94 ± 0.53 | 0.051 |
| Phosphorus (mmol/l) | 1.18 ± 0.22 | 1.07 ± 0.21 | <0.001 | 1.16 ± 0.21 | 1.11 ± 0.22 | 0.008 |
| 25 (OH)D  (ng/ml) | 19.06 (13.8 - 25.36) | 17.15 (11.29 - 23.24) | 0.042 | 17.11 (12.89 - 22.71) | 14.82 (10.75 - 23.02) | 0.895 |
| N-Mid Osteocalcin (ng/ml) | 17.9 (13.35 - 23.97) | 14.35 (10.72 - 20.21) | 0.017 | 42.81 (32.14 - 59.23) | 41.52 (29.87 - 54.13) | 0.138 |
| N-Terminal Propeptide of Type I Collagen (ng/ml) | 47.17 (35.39 - 63.51) | 35.30 (25.37 - 58.01) | 0.019 | 0.55 (0.36 - 0.71) | 0.6 (0.38 - 0.77) | 0.271 |
| Beta-C cross-linking peptide (ng/ml) | 0.48 (0.36 - 0.63) | 0.50 (0.33 - 0.7) | 0.959 | 53.25 (45.18 - 67.00) | 50.40 (42.00 - 61.90) | 0.353 |

Abbreviations: ASMI: Appendicular Skeletal Muscle Index, BMI: Body Mass Index, WWI: weight-adjusted-waist index, FPG: fasting blood glucose, HbA1c: glycosylated hemoglobin, CK: Creatine Kinase, TC: total cholesterol, TG: total triglyceride, HDL-C: high-density lipoprotein cholesterol, LDL-C: low-density lipoprotein cholesterol, VLDL-C: very-low-density lipoprotein cholesterol. 25(OH)D: 25-Hydroxy Vitamin D.

**Table S3 Baseline characteristics in male**

| Variables | Training Cohort  ( n = 160) | Validation Cohort  ( n = 74 ) | P |
| --- | --- | --- | --- |
| Age (year) | 60.60 ± 12.39 | 63.62 ± 13.09 | 0.090 |
| Duration of Diabetes (year) | 6.00 (2.25 - 10.00) | 7.00 (2.00 - 10.00) | 0.898 |
| Use of hypoglycaemic drugs, n (%) | 139 (86.90) | 61 (82.40) | 0.370 |
| Smoking status, n (%) | 65 (40.60) | 30 (40.50) | 0.990 |
| Drinking status, n (%) | 28 (17.50) | 9 (12.20) | 0.298 |
| Hypertension, n (%) | 66 (41.30) | 31 (41.90) | 0.926 |
| Duration of Hypertension (year) | 0.00 (0.00 - 1.00) | 0.00 (0.00 - 1.00) | 0.920 |
| ASMI (kg/m^2^) | 7.00 ± 1.09 | 6.71 ± 1.07 | 0.061 |
| Handgrip strength (kg) | 25.01 ± 1.54 | 24.9 ± 1.63 | 0.825 |
| Gait speed (m/s) | 0.83 ± 0.09 | 0.85 ± 0.12 | 0.672 |
| Height (cm) | 163.35 ± 6.58 | 164.16 ± 6.91 | 0.391 |
| Weight (cm) | 60.11 ± 9.83 | 59.36 ± 9.10 | 0.578 |
| BMI (kg/m^2^) | 22.49 ± 3.17 | 22.01 ± 3.03 | 0.276 |
| Waist Circumference (cm) | 82.72 ± 8.77 | 82.67 ± 9.43 | 0.967 |
| WWI (cm/√kg) | 10.71 ± 0.74 | 10.74 ± 0.89 | 0.805 |
| Systolic Blood Pressure (mmHg) | 135.01 ± 19.66 | 136.26 ± 20.97 | 0.660 |
| Diastolic Blood Pressure (mmHg) | 79.06 ± 11.12 | 78.43 ± 10.92 | 0.686 |
| Heart Rate (beats/min) | 80.32 ± 11.77 | 81.92 ± 13.13 | 0.356 |
| FPG (mmol/l) | 7.91 (5.54 - 12.10) | 7.65 (5.36 - 12.35) | 0.582 |
| HbA1c (%) | 10.01 ± 2.68 | 9.74 ± 2.79 | 0.497 |
| White Blood Cell (×10^9^/L） | 6.68 (5.48~8.43) | 6.57 (4.91~7.85) | 0.159 |
| Neutrophil (×10^9^/L） | 4.06 (3.07 - 5.51) | 4.21 (3.14 - 5.47) | 0.454 |
| Lymphocyte (×10^9^/L） | 1.80 ± 0.73 | 1.60 ± 0.84 | 0.076 |
| Hemoglobin (g/l) | 132.56 ± 21.62 | 126.51 ± 22.77 | 0.054 |
| Total Bilirubin (μmol/l) | 10.23 ± 4.76 | 10.70 ± 5.63 | 0.612 |
| Direct Bilirubin (μmol/l) | 3.30 (2.30 - 4.53) | 3.40 (2.53 - 5.78) | 0.266 |
| Indirect Bilirubin (μmol/l) | 6.20 (4.20 - 8.83) | 5.65 (3.68 - 7.78) | 0.407 |
| Total Protein (g/l) | 64.58 ± 7.68 | 64.38 ± 7.11 | 0.881 |
| Albumin (g/l) | 38.00 ± 5.43 | 37.12 ± 4.93 | 0.238 |
| Globulin (g/l) | 27.09 ± 6.08 | 27.85 ± 5.95 | 0.494 |
| Alanine Aminotransferase (U/l) | 21.00 (14.00 - 35.00) | 23.00 (16.00 - 40.00) | 0.638 |
| Aspartate Aminotransferase (U/l) | 22.00 (17.00 - 30.00) | 20.00 (16.00 - 30.00) | 0.706 |
| Lactate Dehydrogenase (U/l) | 179.00 (157.00 - 215.00) | 180.00 (155.25 - 205.50) | 0.758 |
| CK (U/l) | 66.00 (42.00 - 100.50) | 57.00 (43.00 - 96.00) | 0.605 |
| Urea (mmol/l) | 5.28 (4.24 - 7.10) | 5.50 (4.48 - 6.76) | 0.573 |
| Creatinine (μmol/l) | 65.60 (54.10 - 78.90) | 65.10 (54.85 - 78.65) | 0.695 |
| Uric Acid (μmol/l) | 299.00 (239.20 - 361.00) | 310.80 (243.75 - 374.3) | 0.376 |
| TG (mmol/l) | 1.17 (0.81 - 2.04) | 1.08 (0.8 - 1.56) | 0.390 |
| TC (mmol/l) | 4.62 ± 1.53 | 4.54 ± 1.92 | 0.734 |
| HDL - C (mmol/l) | 1.12 (0.85 - 1.42) | 1.12 (0.94 - 1.41) | 0.617 |
| LDL - C (mmol/l) | 2.85 ± 1.07 | 2.65 ± 1.01 | 0.183 |
| VLDL - C (mmol/l) | 0.45 (0.31 - 0.64) | 0.44 (0.33 - 0.57) | 0.821 |
| Sodium (mmol/l) | 140.72 ± 3.49 | 140.41 ± 3.15 | 0.528 |
| Potassium (mmol/l) | 4.02 ± 0.55 | 4.00 ± 0.49 | 0.821 |
| Phosphorus (mmol/l) | 1.13 ± 0.22 | 1.10 ± 0.23 | 0.365 |
| Calcium (mmol/l) | 2.21 ± 0.16 | 2.19 ± 0.15 | 0.595 |
| 25 (OH)D (ng/ml) | 18.84 (13.9 - 24.86) | 17.26 (11.16 - 24.32) | 0.330 |
| N-Mid Osteocalcin (ng/ml) | 16.34 (12.36 - 21.29) | 14.32 (9.68 - 23.17) | 0.242 |
| N-Terminal Propeptide of Type I Collagen (ng/ml) | 41.51 (27.79 - 59.53) | 35.39 (26.11 - 66.27) | 0.329 |
| Beta-C cross-linking peptide (ng/ml) | 0.48 (0.35 - 0.64) | 0.49 (0.35 - 0.70) | 0.187 |

Abbreviations: BMI: Body Mass Index, WWI: weight-adjusted-waist index, FPG: fasting blood glucose, HbA1c: glycosylated hemoglobin, CK: Creatine Kinase, TC: total cholesterol, TG: total triglyceride, HDL-C: high-density lipoprotein cholesterol, LDL-C: low-density lipoprotein cholesterol, VLDL-C: very-low-density lipoprotein cholesterol. 25 (OH)D: 25-Hydroxy Vitamin D.

**Table S4 Baseline characteristics in female**

| Variables | Training Cohort  ( n = 459) | Validation Cohort  ( n = 154) | P |
| --- | --- | --- | --- |
| Age (year) | 66.57 ± 8.67 | 67.5 ± 8.56 | 0.228 |
| Duration of Diabetes (year) | 8.00 (3.00 - 13.00) | 9.00 (2.00 - 12.00) | 0.345 |
| Use of hypoglycaemic drugs, n (%) | 371 (85.90) | 152 (84.00) | 0.544 |
| Smoking status, n (%) | 2 (0.50) | 2 (1.10) | 0.368 |
| Drinking status, n (%) | 0 (0.00) | 0 (0.00) | NA |
| Hypertension, n (%) | 234 (54.20) | 103 (56.90) | 0.534 |
| Duration of Hypertension (year) | 1.00 (0.00 - 3.00) | 1.00 (0.00 - 1.00) | 0.712 |
| ASMI (kg/m^2^) | 5.99 ± 0.94 | 6.08 ± 0.88 | 0.248 |
| Handgrip strength (kg) | 17.17 ± 0.98 | 17.23 ± 1.31 | 0.833 |
| Gait speed (m/s) | 1.01 ± 0.10 | 0.93 ± 0.08 | 0.425 |
| Height (cm) | 152.14 ± 5.95 | 152.2 ± 5.61 | 0.907 |
| Weight (cm) | 54.62 ± 9.08 | 55.27 ± 9.42 | 0.421 |
| BMI (kg/m^2^) | 23.59 ± 3.68 | 23.84 ± 3.77 | 0.453 |
| Waist Circumference (cm) | 84.43 ± 10.09 | 85.19 ± 10.09 | 1.421 |
| WWI (cm/√kg) | 11.47 ± 0.84 | 11.5 ± 0.94 | 0.635 |
| Systolic Blood Pressure (mmHg) | 140.16 ± 21.17 | 141.84 ± 21.20 | 0.371 |
| Diastolic Blood Pressure (mmHg) | 76.73 ± 10.73 | 77.17 ± 10.94 | 0.650 |
| Heart Rate (beats/min) | 80.10 ± 11.98 | 80.10 ± 13.66 | 0.998 |
| FPG (mmol/l) | 7.78 (5.67 - 10.73) | 7.22 (5.35 - 10.27) | 0.136 |
| HbA1c (%) | 9.18 ± 2.43 | 2.43 ± 8.86 | 0.153 |
| White Blood Cell (×10^9^/L） | 6.62 (5.32 - 8.30) | 5.95 (5.00 - 7.78) | 0.012 |
| Neutrophil (×10^9^/L） | 3.97 (2.93 - 5.35) | 3.67 (2.65 - 4.97) | 0.015 |
| Lymphocyte (×10^9^/L） | 1.86 ± 0.70 | 1.88 ± 0.79 | 0.865 |
| Hemoglobin (g/l) | 122.32 ± 16.44 | 122.61 ± 18.13 | 0.850 |
| Total Bilirubin (μmol/l) | 10.09 ± 5.28 | 9.99 ± 5.09 | 0.857 |
| Direct Bilirubin (μmol/l) | 2.90 (2.20 - 3.90) | 2.90 (2.20 - 4.30) | 0.637 |
| Indirect Bilirubin (μmol/l) | 5.90 (4.33 - 7.90) | 5.80 (4.20 - 8.70) | 0.797 |
| Total Protein (g/l) | 66.59 ± 6.66 | 67.52 ± 7.12 | 0.211 |
| Albumin (g/l) | 38.45 ± 4.80 | 38.28 ± 4.39 | 0.690 |
| Globulin (g/l) | 28.03 ± 4.49 | 29.1 ± 6.05 | 0.051 |
| Alanine Aminotransferase (U/l) | 17.00 (13.00 - 26.00) | 18.00 (13.00 - 25.00) | 0.896 |
| Aspartate Aminotransferase (U/l) | 20.00 (16.00 - 26.00) | 19.50 (16.00 - 25.00) | 0.384 |
| Lactate Dehydrogenase (U/l) | 187.00 (159.00 - 212.75) | 180.00 (159.00 - 209.00) | 0.598 |
| CK (U/l) | 57.00 (39.50 - 81.50) | 58.00 (39.00 - 89.25) | 0.413 |
| Urea (mmol/l) | 5.17 (4.01 - 6.51) | 5.34 (4.05 - 6.65) | 0.405 |
| Creatinine (μmol/l) | 52.75 (43.98 - 66.00) | 52.10 (45.00 - 67.00) | 0.841 |
| Uric Acid (μmol/l) | 275.25 (208.85 - 350.63) | 280.5 (221.33 - 346.13) | 0.816 |
| TG (mmol/l) | 1.59 (1.01 - 2.23) | 1.39 (1.03 - 2.13) | 0.197 |
| TC (mmol/l) | 4.93 ± 1.35 | 4.68 ± 1.21 | 0.032 |
| HDL - C (mmol/l) | 1.16 (0.96 - 1.43) | 1.14 (0.94 - 1.45) | 0.780 |
| LDL - C (mmol/l) | 2.94 ± 1.05 | 2.81 ± 1.06 | 0.155 |
| VLDL - C (mmol/l) | 0.56 (0.38 - 0.88) | 0.56 (0.40 - 0.85) | 0.842 |
| Sodium (mmol/l) | 141.45 ± 3.25 | 141.89 ± 2.74 | 0.110 |
| Potassium (mmol/l) | 4.00 ± 0.51 | 4.03 ± 0.53 | 0.562 |
| Phosphorus (mmol/l) | 1.14 ± 0.22 | 1.17 ± 0.20 | 0.071 |
| Calcium (mmol/l) | 2.23 ± 0.15 | 2.24 ± 0.14 | 0.389 |
| 25 (OH)D (ng/ml) | 18.13 (13.06 - 23.50) | 17.46 (12.14 - 23.01) | 0.460 |
| N-Mid Osteocalcin (ng/ml) | 17.37 (12.22 - 22.98) | 15 (11.95 - 22.36) | 0.360 |
| N-Terminal Propeptide of Type I Collagen (ng/ml) | 42.39 (31.73 - 55.83) | 41.86 (32.52 - 61.25) | 0.758 |
| Beta-C cross-linking peptide (ng/ml) | 0.56 (0.38 - 0.73) | 0.54 (0.33 - 0.72) | 0.418 |

Abbreviations: BMI: Body Mass Index, WWI: weight-adjusted-waist index, FPG: fasting blood glucose, HbA1c: glycosylated hemoglobin, CK: Creatine Kinase, TC: total cholesterol, TG: total triglyceride, HDL-C: high-density lipoprotein cholesterol, LDL-C: low-density lipoprotein cholesterol, VLDL-C: very-low-density lipoprotein cholesterol. 25 (OH)D: 25-Hydroxy Vitamin D.
